# Levitation of posteriorly dislocated intraocular lens: I.V. catheter connected to the vitreotome aspiration

**DOI:** 10.3389/fopht.2025.1547363

**Published:** 2025-03-13

**Authors:** Ruiping Gu, Yue Guo, Yuan Zong, Rui Jiang, Zhongcui Sun

**Affiliations:** ^1^ Department of Ophthalmology, Eye and ENT Hospital of Fudan University, Shanghai, China; ^2^ Key Laboratory of Myopia and Related Eye Diseases, National Health Commission (NHC), Shanghai, China; ^3^ Key Laboratory of Myopia and Related Eye Diseases, Chinese Academy of Medical Sciences, Shanghai, China

**Keywords:** posteriorly dislocated intraocular lens, I.V. catheter, vitreotome aspiration, vitrectomy 23-gauge, intraocular forceps

## Abstract

**Background:**

To introduce a new, simple, and affordable technique that uses a 22G intravenous (I.V.) catheter connected to the vitreotome aspiration to lift the intraocular lens (IOLs) off the retina.

**Methods:**

This retrospective, non-comparative, single surgeon, interventional, consecutive case series examined 4 patients (4 eyes) who underwent the surgical procedure from March 12 and October 22, 2023. Reliability, reproducibility, and intraoperative and postoperative complications of the technique were analyzed.

**Results:**

Four patients presenting with posteriorly dislocated IOLs were included. After a complete 23G vitrectomy under wide-angle viewing system or high magnification contact lens, the 22G I.V. catheter was connected to the vitreotome aspiration and active aspiration was applied. When the IOLs were lifted towards the posterior chamber by continuous vacuum aspiration, they were be safely grasped using intraocular forceps and reposited with scleral fixation suturing or removed through a limbal incision. None of the IOLs fell during active aspiration.

**Conclusion:**

Connection of I.V. catheter with vitreotome aspiration to lift the IOLs off the retina was a new, simple, safe, and affordable technique.

## Background

Dislocation of a posterior chamber intraocular lens IOL into the vitreous cavity is an uncommon but significant complication (1, 2). Various methods have been described to manage the dislocated IOL (3, 4). One critical step is lifting the IOL off the retina into the posterior chamber. Traditional techniques involve grasping the haptic and lift the IOL off the retina using vitreous forceps (5). However, directly grasping the IOL with forceps can easily cause inadvertent retinal damage since the IOL frequently rests on the retinal surface or even in front of the macula. Perfluorocarbon liquid (PFCL) was used to safely float the IOL off the retina and into the position behind the pupil (5). Nevertheless, during the injection of PFCL, the IOL may float across the surface of the PFCL bubble and cause retinal damage. Santos and Roig-Melo and Agarwal et al. described the successful use of sleeve- and sleeveless extrusion cannula to levitate dislocated IOLs (6, 7).

In this study, we introduce a new, simple, and affordable technique that uses 22G (22-gauge) I.V. catheter connected to the vitreotome aspiration to lift the IOL off the retina.

## Methods

This study was a retrospective noncomparative series of consecutive cases done at the Eye and Ear, Nose, and Throat (EENT) Hospital of Fudan University (Shanghai, China) between March 12 and October 22, 2023. The protocols and informed consent forms were approved by the institutional review board of the EENT hospital of Fudan University. Clinical records of patients with posteriorly dislocated IOLs were reviewed. All surgeries were performed by one surgeon (Zhongcui Sun). Informed consents were taken from all the patients. Subjects with less than 6 months of postoperative follow up were excluded.

All the participants underwent preoperative and postoperative ophthalmological examinations, including the best-corrected visual acuity (BCVA) test, dilated indirect slit-lamp biomicroscope examination, intraocular pressure (IOP) test using a non-contact tonometer (Nidek NT400, Nidek Co., Ltd., Aichi, Japan), corneal endothelial cell density count using a non-contact specular microscope (Topcon America Corporation, Paramus, NJ, USA), and axial length (AL) measurement using IOLMaster 700 (version 3.01; Carl Zeiss Meditec, Jena, Germany). Intraoperative conditions were recorded, including types of dislocated IOLs, fall of IOL during removement, or any other intraoperative complications. Postoperative examination focused on BCVA, IOP, and anterior and posterior segment findings, with particular attention to identifying any postoperative complications.

### Surgery techniques

**Figure 1:**
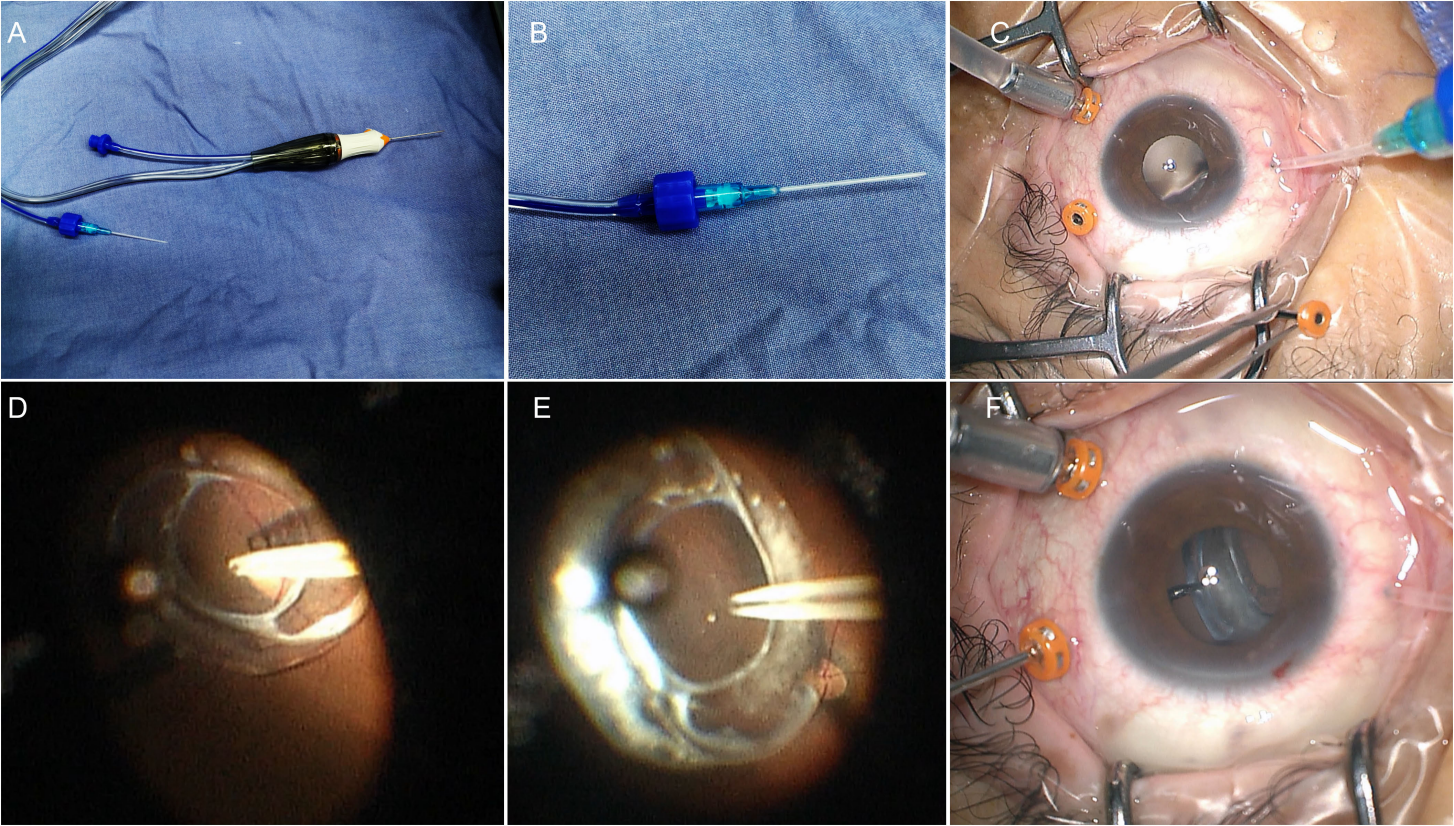
The 22-Gauge I.V. catheter connected to the vitreotome aspiration to lift the IOL off the retina. **(A)** The I.V. catheter connected to the vitreotome aspiration system. **(B)** Magnification of the connection. **(C)** Insert the catheter through the scleral tunnel directly. **(D)** The catheter tip was placed on the center of the anterior surface of the IOL optic. **(E)** Aspirate the IOL from the posterior retina. **(F)** Grasped the IOLs with the forceps in anterior segment.

After a standard three-port pars plane complete vitrectomy, a 22G I.V. catheter was connected to the vitreotome aspiration and the vacuum was set to 300 mm Hg, with the cutting function turned off (**Figures 1A, B**). One of the trocar-cannulas (supertemporal or supranasal) was pulled out and the 22G catheter was directly inserted through the scleral tunnel (**Figure 1C**). Active aspiration was applied only when catheter tip was placed very close to the anterior surface of the IOL optical surface (**Figure 1D**). Vacuum was adjusted by the foot switch and titrated according to the ability to lift the IOL (**Figure 1E**). The IOL was then lifted towards the posterior chamber by continuous vacuum aspiration, grasped by intraocular forceps (**Figure 1F**) and reposited with scleral fixation suturing or removed through a limbal incision. The surgical video was submitted as **Supplementary Material** (**Supplementary Video S1**).

## Results

**Figure 2:**
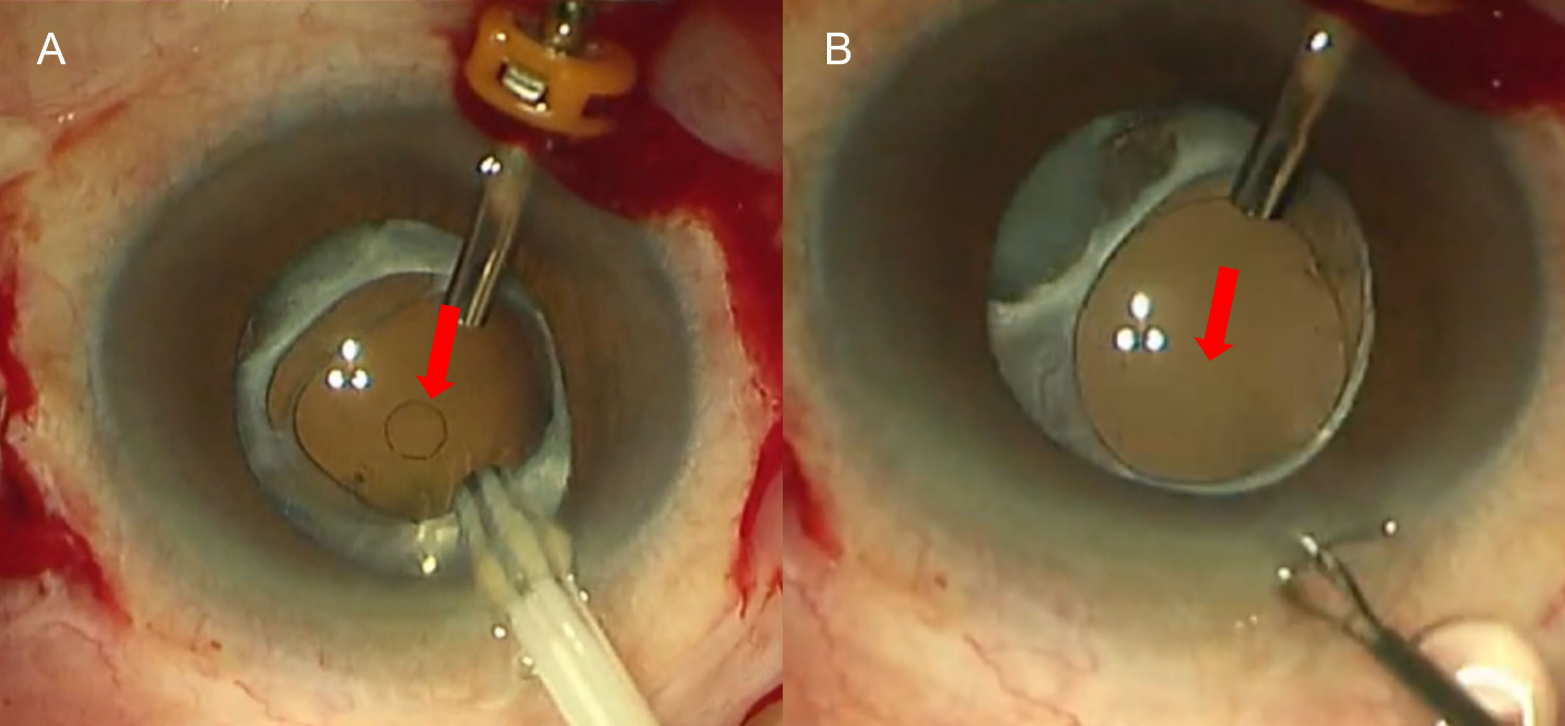
A circular mark on the surface of an IOL during suction and subsequent transparency restoration. **(A)** A circular mark appeared on the surface of the foldable IOL during suction; **(B)** it disappeared a few seconds later. Red arrow indicates the location of the circular mark.

The study included four eyes of four patients (two males and two females; mean age 64.5 ± 6.40 years; range 58-70 years). All patients had a history of one or more times of ocular surgeries and denied any history of ocular trauma. Patients’ characteristics were summarized in **Table 1**. The average AL was 24.58 ± 1.95 mm, preoperative mean IOP was 15 ± 0.82 mmHg, and preoperative mean BCVA was 0.69 ± 0.46 (log MAR). Among the four dislocated IOLs, one was a plate-haptic IOL, two were one-piece foldable IOLs, and one was a three-piece foldable IOL. IOL exchange with sutured scleral fixation of a new IOL was performed in the patient with a plate-haptic IOL. Repositioning with scleral fixation suturing of the primary IOL was performed in the other three patients. A circular mark may appear on the surface of the foldable IOL during suction and soon disappeared, and the IOL returned to transparency (**Figure 2**). None of the IOLs fell during active aspiration. No intraoperative complication was observed during 6-month follow-up. The IOLs remained well-positioned in all four patients. The mean BCVA was 0.50 ± 0.42 (log MAR), and the mean IOP was 17.4 ± 2.50 mmHg.

**Table 1:** Demographics of eyes with 22G (22-gauge) I.V. catheter connected to the vitreotome aspiration levitation of dislocated posterior chamber IOL (vision in logMAR).

Patients	Age/sex	Past medical history in IOL dislocated eye	Axial length (mm)	BCVA Preoperative (logMAR)	IOP Preoperative (mmHg)	Type of IOL levitated	IOL repositioning or IOL exchange	BCVA postoperative(logMAR)	IOP postoperative (mmHg)
1	58/F	In 2001, underwent scleral buckling surgery for RRD in the right eye. In 2005, experienced retinal re-detachment in the right eye, and underwent vitrectomy+ phacoemulsification and IOL implantation+ pneumatic retinopexy	22.86	1.30	14	3-piece foldable	Repositioning with sutureless intrascleral fixation	1	17.6mmHg
2	70/M	In 1983, underwent scleral buckling surgery for RRD in left eye In 2001, underwent phacoemulsification and IOL implantation in the left eye. In 2006, underwent vitrectomy with silicon oil tamponade and remove for retinal re-detachment.	26.95	0.70	15	Plate haptic	IOL exchange with sutured scleral fixation	0.7	18mmHg
3	60/F	In 2008, underwent phacoemulsification and IOL implantation in the left eye	25.41	0.22	16	1-piece foldable	Repositioning with sutured scleral fixation	0.22	20mmHg
4	70/M	In 2023, underwent vitrectomy+ phacoemulsification and IOL implantation for BRVO induced vitreous hemorrhage	23.1	0.52	15	1-piece foldable	Repositioning with sutured scleral fixation	0.1	14mmHg

BCVA, best corrected visual acuity; F, female; IOL, intraocular lens; IOP, intraocular pressure; M, male; RRD, rhegmatogenous retinal detachment; BRVO, branch retinal vein occlusion.

## Discussion

Dislocation of a posterior chamber intraocular lens (IOL) into the vitreous cavity is an uncommon but serious complication (1, 2). Numerous techniques for managing posteriorly dislocated IOLs have been described (3, 4). Intraocular forceps are often used to manipulate dislocated IOLs and are typically the standard treatment in vitreoretinal surgery (5). However, IOLs can be slippery and difficult to grasp, especially plate haptic IOLs. Accidental iatrogenic retinal damage during lifting an IOL from the retinal surface are not uncommon with this approach.

PFCL is used to protect the posterior retina from damage and to facilitate the anterior dislocation of luxated IOLs (8, 9). High density of PFCL allows for levitating the IOL into the pupillary plane. Low viscosity of PFCL facilitates easy aspiration and injection in a 23-gauge vitrectomy system. However, possible retained PFCL causes ocular toxicity, including uncontrolled intraocular pressure (IOP), corneal epithelial toxicity, and decreased retinal sensitivity.

Santos, Roig-Melo, and Agarwal et al. (6, 7) described the successful use of sleeve and sleeveless extrusion cannulas connected to the vitrectome vacuum to elevate dislocated IOLs. The vacuum created by the extrusion cannula is strong enough to hold the optic surface of an IOL. No pressure is exerted on the IOL while trying to create suction, and no passive suction flow to move the IOL is attempted. This prevents any uncontrolled movement of the IOL that could be detrimental to the retina. The linear control of the foot pedal allows for precise regulation of the vacuum during IOL levitation. Thus, lifting the IOL with vacuum aspiration appears to be safer than intraocular forceps and avoids the need for PFCL.

The extrusion cannula is primarily used for internal drainage of subretinal fluid in eyes with rhegmatogenous retinal detachments and for removing hemorrhage, gas, or silicone oil in the subretinal space (10). However, not all eye centers are supplied with the costly extrusion cannula, and not all extrusion cannulas can be connected to vitrectomy vacuum aspiration system. We introduce an easy, simple, and affordable technique that uses a 22G intravenous I.V. catheter connected to the vitreotome aspiration system. The 22G I.V. catheter, a common hospital supply, is easily accessible and inexpensive. The catheter is disposable and prevents iatrogenic infection. The catheter can connect to the vitreotome aspiration perfectly. Additionally, the 22G I.V. catheter can smoothly pass through a 23G scleral tunnel without further enlargement. If the catheter is inserted through a corneal limbal incision, the incision is small and self-sealing. The length of I.V. catheter is 31mm, long enough to reach the IOL above the posterior retina, regardless of the entrances through a limbal incision or a scleral incision. This technique was reproducible in all the cases and was effective for dislocation of any type of IOL especially the plate haptic IOLs which are difficult to grasp using intraocular forceps. In case of a foldable IOL, a circular mark will be left on the surface of the IOL during suction. The mark will soon disappear and the IOLs will return transparency. Although this surgical technique was controllable, the surgeon had to ensure that suction pressure was maintained throughout the entire suction process. This was especially crucial when grasping the IOL haptic with the left hand—any interruption in suction pressure could have caused the IOL to dislocate again, posing a risk of iatrogenic retinal injury. Additionally, since the core principle of this technique relied on suction pressure, a thorough vitrectomy had to be performed beforehand. If any vitreous was inadvertently aspirated during suction, it could have led to retinal tears or even retinal dialysis. Therefore, a comprehensive peripheral retinal examination needed to be conducted before concluding the surgery.

In conclusion, a 22G I.V. catheter connected to vitrectome aspiration can be an easy and affordable method for removal of dislocated IOLs.

## Data Availability

The original contributions presented in the study are included in the article/**Supplementary Material**. Further inquiries can be directed to the corresponding author.
